# Occupational Safety and Health Vulnerability among Brick Factory Workers in Dhading District, Nepal

**DOI:** 10.29024/aogh.2313

**Published:** 2018-08-31

**Authors:** Dinesh Rupakheti, Pranil Man Singh Pradhan, Prem Basel

**Affiliations:** 1 Department of Community Medicine and Public Health, Institute of Medicine, Tribhuvan University, Kathmandu, NP

## Abstract

**Background:**

Occupational safety and health vulnerability considers multiple dimensions, such as hazard, policy/procedure to protect workers, workers’ awareness and their empowerment to participate in injury prevention. This study attempts to bridge the inadequate knowledge regarding the factors associated with occupational safety and health vulnerability in brick factories.

**Objectives:**

To identify the status and factors associated with occupational safety and health vulnerability among brick factory workers in Dhading district.

**Methods:**

A cross-sectional descriptive study was carried out in five brick kilns of Dhading district. A two-stage cluster sampling method was used; at the first stage, probability proportionate to sample size was used to select the brick factories and simple random sampling was used to select participants from each selected brick factory. A total of 201 workers with at least a year of experience and who had worked over the last year in brick factories were included in the study. The data was collected through face-to-face interviews using a structured questionnaire. Vulnerability was defined as exposed to hazards and having inadequate policies, procedures, awareness and empowerment. Pearson Chi-square test was used to examine the relationship between vulnerability and demographic, occupational and workplace characteristics.

**Results:**

Four-fifths of the participants experienced occupational safety and health vulnerability. All participants experienced policy/procedure vulnerability; four-fifths experienced hazard vulnerability and about two-thirds experienced awareness and empowerment vulnerability. Younger, nonnative immigrants, workers carrying bricks out of a chimney and workers from small-sized workplaces experienced higher odds of vulnerability across all domains and overall vulnerability.

**Conclusion:**

Occupational safety and health vulnerability was very high among the brick factory workers. Young workers, non-native immigrant workers, workers carrying cooked bricks out of a chimney and workers from small-sized workplace were found to be more vulnerable.

## Introduction

Occupational safety and health (OSH) is the science of anticipation, recognition, evaluation and control of hazards arising in or from the workplace, which could impair the health and well-being of workers, and also impact the surrounding communities and the environment [1]. It is a cross-disciplinary area concerned with protecting the safety, health and welfare of people engaged in work or employment.

Occupational injury, illness and workplace facilities are important public health concerns. The International Labor Organization (ILO) estimates that more than 2.3 million deaths per year occur as a result of occupational accidents or work-related diseases. About 317 million accidents occur on the job annually; an economic cost is 1.8% and 6% of Gross Domestic Product (GDP) in country estimates, being average 4% [2]. In Nepal, 20,000 workers annually suffer from accidents at the workplace, which leads to about 200 lives lost. The OSH situation is even worse in the informal sector which include about 70% of the economically active population of Nepal. Although ILO has maintained and developed a system of international labor standards since 1919, which aims at promoting opportunities for decent and productive work in conditions of freedom, equity, security and dignity, the concept of OSH is relatively new in Nepal, and very few industries achieve even the basic occupational standards [3].

The Nepalese economy is characterized by a dominant agricultural sector employing 70% of the population and providing 37% of GDP. Nowadays, the position of the service sector, construction and transportation is progressively increasing [4]. The brick industry is one of the oldest industries in Nepal, and it falls under small/cottage industry group despite large investment. It is a seasonal industry, with a seasonal employment opportunity. With the expansion of urban areas, the brick industry is also rapidly growing [4]. There are about 705 brick kilns operating in Nepal, employing more than 175,000 workers [5].

Brick workers in Nepal are the most marginalized of the unskilled workers, often bonded by debt and forced to work for insufficient wages. The brick industries employ migrant and seasonal laborers who have no link to local government or representation by workers’ associations and are situated mostly in suburban areas [6]. Brick kilns in Nepal use firewood and coal as fuels to bake the bricks, whereas the kilns may or may not operate with chimneys. The laborers of the kiln are mostly from the agricultural sector and are recruited through Naikes (the person who provides workers for the brick industries), brick kiln owners or self-approach. Four main activites take place in the brick factory. These are: preparing mud and laying brick, barrying raw bricks to kiln, working inside the chimney and carrying out cooked brick. Work inside the chimney includes spreading rubbish over the brick, firing the furnace, controlling the fire and flame and clearing the hole. Human laborers carry the baked brick. One Naike controls about 25–30 brick laborers [4].

Major hazards posed to brick kiln workers are chemical, physical, biological, psychosocial and ergonomic. Chemical hazards include exposure to brick dust, silica, carbon monoxide (CO), sulfur dioxide (SO_2_), fluoride compounds and nitrogen oxides (NO_X_). The workers are also exposed to burnt mud dust mixed with coal and cooked brick. Physical hazards include heat stress and excessive exposure to noise while working in the furnace. Biological hazards are contact with soil, insects and animals. Lifting heavy weights, improper posture and repetitive movements are ergonomic hazards that can lead to chronic musculoskeletal problems. Psychosocial hazards are high levels of stress, low self-esteem and abuse at work [7,8].

Many studies have documented that the burden of occupational injury and illness is not equally distributed across the labor force [9,10,11,12,13]. Not all workers have the same risk of being injured at work, even when they are in the same industry or have the same job. Defining vulnerability only in terms of individual demographic or job or workplace characteristics is insufficient and unrealistic, as it does not adequately consider how the particular circumstances of the workers contribute to their occupational health and safety risk. In addition to differential hazard exposure, social factors such as race, class and gender; economic trends such as the growth of the temporary workforce; and organizational factors such as business size can make some workers more vulnerable than others to workplace illness or injury [11].

The vulnerability assessment should consider multiple dimensions, such as hazard, policy/procedure to protect workers, workers’ awareness and their empowerment to participate in injury prevention. However, there is a knowledge gap regarding vulnerability disparities in relation to these factors.

This study measured vulnerability comprehensively based on four domains. The analysis of variation in these domains helps to identify which domain is to be addressed to minimize overall vulnerability in the brick factory. This study measured OSH vulnerability and examined its relationship with demographic, job and workplace characteristics. Examining how diverse sub-populations in the brick factories are affected by each type of vulnerability differently helps to develop targeted primary prevention strategies to reduce vulnerability. This study aims to provide supportive evidence to inform policy and practical interventions that will protect the health, safety and well-being of brick factory workers and guide future courses of action.

## Methods

### Tools and techniques

Data were collected using a 27-question survey tool developed by Peter Smith et al., Institute for Work and Health, Canada, in 2013, which was designed to improve measurement and evaluation of how workplace context impacts individual risk of workplace injury or illness [9]. The validity of the tool was assessed by consultation with occupational health experts and pretested before application. Internal consistency of the tool was ascertained by pretesting in 10% of the sample size. Cronbach’s alpha was calculated and found to be sufficient (α = 0.8). In the first three sections, questions related to demographic, occupational and workplace characteristics were included whereas the rest of the four sections included information on respondents’ exposure to workplace hazards and on the availability of three types of resources designed to mitigate the impact of exposure to these hazards. The three mitigation resources explored were (a) workplace level policies and procedures; (b) worker awareness of occupational hazards and rights and responsibilities; and (c) worker empowerment to participate in injury prevention.

Data were collected from 205 participants using face-to-face interviews. Finally 201 participants were considered for analysis with a response rate of 98.5 percent. The brick factory workers working for at least one year were included in the study. Administrative staff were excluded from the study. Ethical approval was obtained from the Institutional Review Board at Institute of Medicine, Kathmandu, Nepal.

### Variables

Demographic variables included age classified into four categories <25 years, 25–34 years, 35–44 years and 45 years and above); gender (male or female); origin of participants (Nepal or outside of Nepal); and literacy status (literate or not). Occupational variables included work experience (1–2 years, 3–5 years, 6–10 years and more than 10 years), nature of work (preparing mud and lying bricks, carrying raw bricks to the kiln, working inside the chimney and carrying cooked bricks for transportation) employment relationship (temporary versus permanent). Workplace characteristics was limited to workplace size (≤50 workers or >50 workers).

In this study, the respondents were considered as occupationally vulnerable if they were exposed to both workplace hazards and experienced inadequate resources to mitigate the effects of hazard exposure. Four types of vulnerability (three specific and one overall) were measured. In order to determine the workplace hazard exposure, participants were asked nine questions on how often they experience hazards such as excessive noise, repetitive motions, and prolonged standing. The seven-level response scale ranging from never to every day was used. Individuals were considered exposed if they reported experiencing two or more of the nine hazards weekly or more often, or if they reported weekly or more frequent exposure to either work involving lifting or carrying 20 kg at least 10 times a day, working at heights greater than 2 meters, working with hazardous substances such as chemicals, flammable liquids and gases, or experiencing bullying or harassment.

The adequacy of three types of workplace resources designed to mitigate the effects of hazard exposure was measured by level of agreement (strongly agree, agree, disagree, strongly disagree) with a series of statements. To evaluate adequacy of OSH policy and procedures, seven statements were used about the existence and implementation of workplace systems such as safety training and accident investigations and the presence of an OSH committee or representative. Likewise, six statements on workers’ knowledge of rights and responsibilities and job-specific safety precautions were asked to measure adequacy of worker awareness of OSH rights and responsibilities. Five questions about comfort voicing safety concerns, participation in health and safety improvements etc. were asked to measure the worker empowerment for engaging in health and safety prevention. Within each of the three types of mitigation resources (policies and procedures, awareness and empowerment), respondents were considered to have inadequate access if they disagreed (disagree or strongly disagree) with one or more of the survey statements.

Four dichotomous vulnerability outcomes were created based on exposure to hazards and adequacy of mitigation resources. Individuals were defined as having one of three specific types of vulnerability—policies and procedures, awareness, and empowerment—if they were exposed to hazards on the job and were classified as “inadequate” on the corresponding mitigation resource. Overall vulnerability was defined as exposure to hazards plus inadequacy of any of the three mitigation resources.

## Result

More than four-fifths (80.6%) of the participants were exposed to overall OSH vulnerability due to hazard exposure in conjunction with either inadequate OSH policies/procedures; or inadequate awareness of the workplace rights, responsibilities and hazards; or inadequate empowerment to ensure a safe work setting. Besides overall vulnerability, this study also examined four specific types of vulnerability. All the participants were found to experience policy and procedure vulnerability. The implementation of policy and the safety procedure was reported nil in brick factories. Four-fifths (80.6%) were experiencing hazard vulnerability. Inadequate awareness and inadequate empowerment were reported by 59.2% and 63.2% of the participants respectively (Table 1).

**Table 1:** Distribution of hazard exposure, inadequate awareness (AW) and inadequate empowerment (EM) across demographic, occupational and workplace characteristics (n = 201).

	Exposure to hazard		Inadequate AW		Inadequate EM	

	%	p value	%	p value	%	p value

**Vulnerability**	80.6		59.2		63.2	
**Gender**						
Male	81.3	0.873	64.1	0.338	71.9	0.081
Female	80.3		56.9		59.1	
**Age**						
<25 years	88.9	0.015	66.7	0.038	74.4	.002
25–34 years	83.3		66.7		69.4	
35–44 years	71.4		51.4		45.7	
45 or more years	67.5		42.5		47.5	
**Location of birth**						
Nepal	72.2	0.036	51.4	0.014	55	.009
Outside Nepal	87		68.5		72.8	
**Literacy status**						
Literate	81.6	0.645	48	<.001	56.8	.016
Illiterate	78.9		77.6		73.7	
**Work experience in brick Kiln**						
1–2 years	85.2	0.21	77.8	<.001	81.5	<.001
3–5 years	84.3		70.6		78.4	
6–10 years	70.6		56.9		62.7	
>10 years	82.2		26.7		24.4	
**Nature of work**						
Preparing mud & lying brick	64.7	0.01	47.1	<.001	45.1	<.001
Carrying raw bricks to kiln	86.3		60.8		72.5	
Working inside chimney	83.7		42.9		53.1	
Carrying out cooked brick	88		86		82	
**Size of workplace**						
<50 workers	86	0.001	63.3	0.041	69.3	.002
>50 workers	64.7		47.1		45.1	

The overall vulnerability was significantly more among young participants, immigrant participants, participants carrying cooked bricks out of the chimney and workers from small-sized workplaces. Illiterate participants experienced nearly fourfold and twofold increases in awareness and empowerment vulnerability, respectively, over their literate counterparts. But the literacy status was statistically not significant to hazard and overall vulnerability (Table 2).

**Table 2:** Unadjusted logistic models for awareness, empowerment and overall vulnerability (n = 201).

	Vulnerability					

	Awareness		Empowerment		Overall	

	Effect	*95%CI*	Effect	*95%CI*	Effect	*95%CI*

**Gender**						
Male	Ref		Ref		Ref	
Female	0.7	0.4, 1.4	0.6	0.3, 1.1	0.9	0.4, 2.0
**Age**						
<25 years	2.7	1.3, 5.8	3.2	1.5, 7.0	3.9	1.5, 9.8
25–34 years	2.7	1.1, 6.9	2.5	0.9, 6.4	2.4	0.8, 7.2
35–44 years	1.4	0.6, 3.6	0.9	0.4, 2.3	1.2	0.5, 3.2
45 or more years	Ref		Ref		Ref	
**Origin**						
Native	Ref		Ref		Ref	
Non-native	2.1	1.2, 3.7	2.2	1.2, 4.0	2.2	1.0, 4.6
**Literacy status**						
Literate	0.3	0.1, 0.5	0.5	0.3, 0.9	1.2	0.6, 2.4
Illiterate	Ref		Ref		Ref	
**Work experience in brick Kiln**						
1–2 years	9.6	3.8, 24.2	13.6	5.2, 35.7	1.3	0.5, 3.7
3–5 years	6.6	2.7, 16.1	11.2	4.3, 29.1	1.1	0.4, 3.4
6–10 years	3.6	1.5, 8.6	5.2	2.1, 12.6	0.5	0.2, 1.4
>10 years	Ref		Ref		Ref	
**Nature of work**						
Preparing mud & lying brick	Ref		Ref		Ref	
Carrying raw bricks to kiln	1.7	0.8, 3.8	3.2	1.4	3.4	1.3, 9.2
Working inside chimney	0.8	0.4, 1.9	1.4	0.6	2.8	1.1, 7.2
Carrying out cooked brick	6.9	2.6, 18.2	5.5	2.2	4	1.4, 11.2
**Size of workplace**						
<50 workers	Ref		Ref		Ref	
>50 workers	0.5	0.3, 0.9	0.4	0.2, 0.7	0.3	0.1, 0.6

## Discussion

These results are consistent with previous studies done in Canada in 2015 and in the USA in 2013, which found that young and immigrant workers were more vulnerable to OSH [10,11]. Youngest workers were found to experience higher vulnerability across three domains viz. hazard, awareness and empowerment and overall OSH vulnerability. Younger workers have lack of job knowledge and skills, lack of job training and lack of job control, which contribute to heightened risk [10,12,13,14]. Individual factors, such as adolescent risk taking and sensation seeking due to the desire to pursue novel and intense experiences, may increase their likelihood of experiencing hazards [15,16,17]. These contribute to heightened risk among younger workers, who might be less likely to recognize hazards, less likely to speak up regarding safety issues [18], and less aware of their legal rights as workers [14,19].

The vulnerability experienced by immigrant workers from India was significantly higher compared to native workers of Nepal across all three domains viz. hazard, awareness and empowerment and overall OSH vulnerability. This finding is similar to earlier research, which suggests that immigrants often have lower levels of OSH awareness and empowerment due to language and cultural barriers [12]. All the immigrants in this study were from India. There is an open border between Nepal and India, and large numbers of labourers come to Nepal from India to work seasonally. They are more vulnerable than the natives due to their low voice for occupational safety and health and low collective bargaining. They are brought and controlled by the Naikes (the person who provides workers to the brick industries) and have very low power to deal with the owner of the factory directly.

Though participants with fewer years of experience were found more vulnerable than those with more experience with respect to awareness and empowerment, no statistically significant association was found between work experience and hazard and overall vulnerability. A similar study in the past has also shown an inverse trend between length of employment and overall occupational vulnerability [20]. Participants having more than ten years of work experience in the brick factory were found to be more vulnerable than those having six to ten years of work experience. This may be due to the fact that most of the workers entered the brick kiln as unskilled workers for laying green bricks. After a certain number of years of experience, they entered as the skilled workers to work inside chimney, which is more hazardous than their previous job. This also may be due to the fact that though workers are more experienced, some type of work is hazardous in itself.

Occupational safety and health vulnerability varies significantly with respect to the nature of work. The workers engaged in carrying cooked bricks out of chimney were found to be more vulnerable across all domains and overall vulnerability. This may be due to the fact that almost all the workers in this type of work were from India. Another reason could be that this work is more hazardous in itself than other types. The workers engaged in carrying the cooked bricks were four times more vulnerable than those workers who prepared mud and laid green bricks. Likewise, those participants who worked inside the chimney and engaged in carrying raw bricks were about three times more vulnerable than those who prepare mud and lay green bricks.

The lower number of workers in a particular nature of job experienced more OSH vulnerability. Those workers who are in few numbers are less likely to be empowered for raising their voices against workplace hazards.

All the participants in the study were vulnerable in terms of policy and procedures. There were no separate policies and procedures for brick factories/establishment to minimize or reduce work-related injuries or to improve work-related safety practices. The issues related to all the industries are addressed in a single act—the Nepal Labour Act, 1991 and Nepal Labour regulation 1993. Like many other countries in the world, Nepal is facing serious problems regarding enactment and enforcement of legislation related to workers in general. There is no comprehensive occupational health and safety law in Nepal. Enactment of Labour Act (1992) and its regulation (1993) is only the legal document that covers safety and health provisions of workers in industrial sector. The Act has prescribed arrangements for garbage management; provision of modern toilets; supply of adequate safe drinking water; provision of appropriate volume of ventilation, condition of light, temperature and sound; protection from dust; smoke, fumes and other impurities; avoidance of overcrowding in any room of the establishment and provision for extinguishing fire. The Act also includes the provision for medical check-ups for the workers at least once a year in the establishments that are prone to hazards. It also suggests a number of preventive measures, such as protection of eyes, protection against chemical hazards and fire, guarding against dangerous machinery, prohibition on lifting heavy load and safety measures for pressure plants. But the Act is silent on which establishments are hazardous and which are safe [21,22].

The study has some limitations. Firstly, the entire study depends upon the interview questionnaire. No measurement of hazard exposure and vulnerability was done using any device. This study was a part of a Master’s level thesis, and the sample size was smaller due to limited resources. However, this study also has a numbers of strengths. Identification of the workers at increased risk of work-related vulnerability is an important strategy in the effort to reduce the burden of workplace morbidity. By identifying hazards that the participants were facing along with policies and procedure, workers’ awareness of health and safety related rights and responsibilities, and individual sense of empowerment, this study highlights how vulnerability differs across various domains. By examining overall vulnerability as well as four distinct types of vulnerabilities, we can better develop specific intervention for a specific group of workers experiencing a specific type of vulnerability. For example, the illiterate individuals might benefit most from awareness-raising interventions. Greater emphasis should be given to educating and raising awareness about occupational health hazards and safety methods to young workers, non-native workers, workers who carry cooked bricks out of the chimney and those from small-sized workplaces. Further research to establish the causality should be conducted in the brick factories to specifically identify the factors that make workers vulnerable.

## Conclusion

The prevalence of occupational safety and health vulnerability is very high among the brick factory workers. Young workers, non-native immigrant workers, workers carrying cooked bricks out from chimney and workers from small-sized workplace were found to be more vulnerable. Knowing how worker subgroups experience different types of vulnerability can help formulate better preventive interventions.

## References

[1] Joshi SK, Shrestha S and Vaidya S. Occupational safety and health studies in Nepal. International Journal of Occupational Safety and Health. 2011; 1(1): 19–26. DOI: 10.3126/ijosh.v1i1.4725

[2] International Labour Organization. Safety and Health at Work. http://www.ilo.org/global/topics/safety-and-health-at-work/lang--en/index.htm. Accessed October 10, 2017.

[3] Gautam R and Prasain J. Current Situation of Occupational Safety and Health in Nepal A Study Report; 2011 https://gefont.org/assets/upload/downloads/Study_OSH_Nepal.pdf. Accessed September 13, 2017.

[4] GEFONT. Nepal: Labour Under the Chimney Kathmandu; 2007 https://www.gefont.org/assets/upload/downloads/brick_klin_report_2008.pdf. Accessed October 9, 2017.

[5] Shah DP. Occupational Safety and Health Assessment in Brick Kilns of Bhaktapur Districts. 2014 (Unpublished Report).

[6] Health Research and Social Development Forum. Brick Kilns in Nepal–A Situation Report; 2016 http://www.herd.org.np/blogs/47. Accessed October 10, 2017.

[7] Thygerson S, Sanjel S and Johnson S. Occupational and environmental health hazards in the brick manufacturing industry in Kathmandu Valley, Nepal. Occup Med Heal Aff. 2016; 4(5).

[8] International Labour Organization. Occupational Health and Safety Assessment of Child Workers in the Brick Industry, Nepal; 2014 http://www.ilo.org/ipec/Informationresources/WCMS_IPEC_PUB_25297/lang--en/index.htm. Accessed October 1, 2017.

[9] Smith P, Saunders R and LaMontagne A. Developing a Framework for Understanding and Measuring Occupational Health & Safety Vulnerability; 2013 http://www.iscrr.com.au/__data/assets/pdf_file/0011/297254/Developing-a-Framework-for-Understanding-and-Measuring-OHS-vulnerability.pdf. Accessed September 1, 2017.

[10] Morgan Lay A, Saunders R, Lifshen M, et al. Individual, occupational, and workplace correlates of occupational health and safety vulnerability in a sample of Canadian workers. Am J Ind Med. 2016; 59(2): 119–28. DOI: 10.1002/ajim.22535 26771101 PMC5019236

[11] NIOSH, ASSE. Overlapping Vulnerabilities: The Occupational Health and Safety of Young Immigrant Workers in Small Construction Firms; 2015 https://www.cdc.gov/niosh/docs/2015-178/pdfs/2015-178.pdf. Accessed October 1, 2017.

[12] Smith PM and Mustard CA. Comparing the risk of work-related injuries between immigrants to Canada and Canadian-born labour market participants. Occupational and Environmental Medicine. 2009; 66(6): 361–367. DOI: 10.1136/oem.2007.038646 18614627

[13] Smith PM and Mustard CA. The unequal distribution of occupational health and safety risks among immigrants to Canada compared to Canadian-born labour market participants: 1993–2005. Safety Science. 2010; 48(10): 1296–1303. DOI: 10.1016/j.ssci.2010.03.020

[14] Laberge M and Ledoux E. Occupational health and safety issues affecting young workers: A literature review. Work. 2011; 39(3): 215–232. 21709358 10.3233/WOR-2011-1170

[15] Spear LP. The adolescent brain and age-related behavioral manifestations. Neuroscience & Biobehavioral Reviews. 2000; 24(4): 417–463. DOI: 10.1016/S0149-7634(00)00014-2 10817843

[16] Steinberg L. Cognitive and affective development in adolescence. Trends in Cognitive Sciences. 2005; 9(2): 69–74. DOI: 10.1016/j.tics.2004.12.005 15668099

[17] Steinberg L. A social neuroscience perspective on adolescent risk-taking. Developmental Review. 2008; 28(1): 78–106. DOI: 10.1016/j.dr.2007.08.002 18509515 PMC2396566

[18] Tucker S and Turner N. Waiting for safety: Responses by young Canadian workers to unsafe work. J Safety Res. 2013; 45: 103–10. DOI: 10.1016/j.jsr.2013.01.006 23708481

[19] NIOSH. Preventing Deaths, Injuries, and Illnesses of Young Workers. NIOSH Alert. https://www.cdc.gov/niosh/docs/2003-128/pdfs/2003128fs.pdf. Accessed October 18, 2017.

[20] Benavides F, Benach J, Muntaner C, Delclos G, Catot N and Amable M. Associations between temporary employment and occupational injury: What are the mechanisms? Occup Environ Med. 2006; 63(6): 416–21. DOI: 10.1136/oem.2005.022301 16497853 PMC2078100

[21] Government of Nepal Ministry of Labour and Employment Department of Labour. Labour Act, 2048 (1992). http://dol.gov.np/ckeditor/kcfinder/upload/files/labor-act-english-2048.pdf. Accessed October 18, 2017.

[22] Government of Nepal Ministry of Labour and Employment Department of Labour. Labour Rules, 2050 (1993) http://dol.gov.np/ckeditor/kcfinder/upload/files/labour-rules-2050-english.pdf. Accessed October 18, 2017.

